# Bottlenecks in the Investigation of Retinal Sterol Homeostasis

**DOI:** 10.3390/biom14030341

**Published:** 2024-03-12

**Authors:** Sriganesh Ramachandra Rao, Steven J. Fliesler

**Affiliations:** 1 Department of Ophthalmology (Ross Eye Institute), Jacobs School of Medicine and Biomedical Sciences, State University of New York, University at Buffalo, Buffalo, NY 14203, USA; fliesler@buffalo.edu; 2 Department of Biochemistry and Neuroscience Graduate Program, Jacobs School of Medicine and Biomedical Sciences, State University of New York, University at Buffalo, Buffalo, NY 14203, USA; 3 Research Service, VA Western New York Healthcare System, Buffalo, NY 14215, USA

**Keywords:** cholesterol, homeostasis, lipid, lipoprotein, retina, retinal degeneration, sterol

## Abstract

Sterol homeostasis in mammalian cells and tissues involves balancing three fundamental processes: de novo sterol biosynthesis; sterol import (e.g., from blood-borne lipoproteins); and sterol export. In complex tissues, composed of multiple different cell types (such as the retina), import and export also may involve intratissue, intercellular sterol exchange. Disruption of any of these processes can result in pathologies that impact the normal structure and function of the retina. Here, we provide a brief overview of what is known currently about sterol homeostasis in the vertebrate retina and offer a proposed path for future experimental work to further our understanding of these processes, with relevance to the development of novel therapeutic interventions for human diseases involving defective sterol homeostasis.

## 1. Introduction

Sterols are ubiquitous cellular constituents throughout the animal kingdom and across phyla. Cholesterol is by far the predominant sterol found in mammalian cells and tissues, including the vertebrate retina, under normal circumstances [1]. However, under circumstances where the normal synthesis of cholesterol is genetically or pharmacologically disrupted, alternative sterols can replace cholesterol, leading to serious, often lethal, pathologies [2,3]. Conversely, the excessive and aberrant deposition of cholesterol, typically in the form of esters and/or oxidized derivatives, in tissues has been associated with different pathologies, such as atherosclerosis [4], age-related macular degeneration (AMD) [5], and diabetic retinopathy [6]. Maintenance of normal steady-state levels and distribution of cholesterol in the vertebrate retina is essential for promoting and maintaining the normal structure and function of the retina. This is achieved via “cholesterol homeostasis”: the balance between local (in the retina per se) cholesterol de novo synthesis, lipoprotein uptake from extraretinal sources (e.g., blood-borne lipoproteins), and its export from the retina (to the blood). The reader is directed to recent, more expansive review articles that have addressed this topic previously [7,8,9]. Here, our intent is to focus on some of the essential facts regarding cholesterol homeostasis and functions in the vertebrate retina, to discuss the current challenges in measuring retinal sterol synthesis and turnover rates, and to point the way toward future experimental approaches that may be applied toward filling in the knowledge gaps that currently exist with regard to this topic.

**Figure 1:**
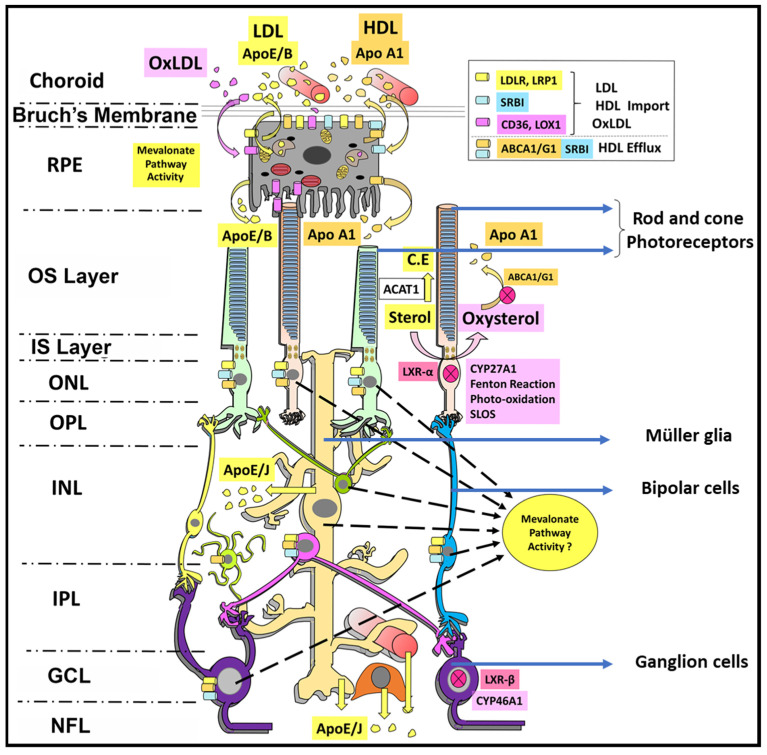
Schematic representation of cholesterol homeostatic processes involved in the vertebrate neural retina. Retinal pigment epithelium (RPE) cells and Müller glia may serve as major hubs for lipoprotein efflux and uptake from/to the neural retina. They also may play a key role in intraretinal distribution of lipoproteins to maintain neuronal sterol demands [8].

**Figure 2:**
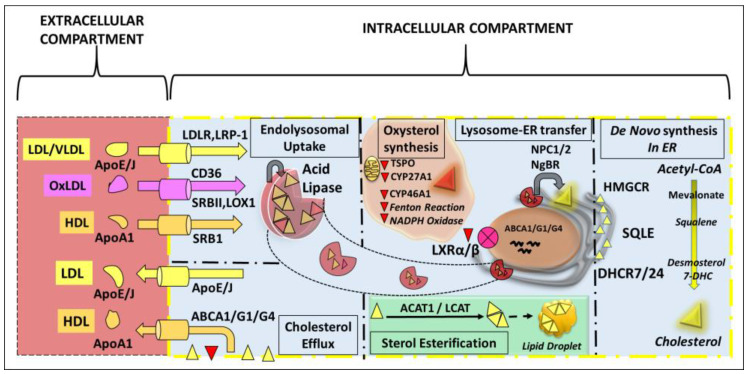
A simplified schematic of intracellular cholesterol homeostasis mechanisms in a “generic” mammalian cell. Cholesterol de novo synthesis occurs in the endoplasmic reticulum (ER) through the mevalonate pathway. ApoE/J-containing lipoproteins are taken up by cells through receptor-mediated endocytosis, followed by lysosome–ER exchange of free cholesterol. Enzymatic and non-enzymatic mechanisms are involved in the generation of hydroxylated and oxidized cholesterol, to enable easy sterol efflux from the cell. An ATP-binding cassette (ABC) transporter system is involved in cellular cholesterol efflux, by sequestering sterols and oxysterols into ApoA1-containing HDL particles. Cells also may sequester cholesterol (and its esters) into lipid droplets.

A summary schematic depicting the localization of the various molecular players (enzymes, receptors, transporters) involved in cholesterol homeostasis in the retina is shown in Figure 1. Additionally, Figure 2 is a simple schematic diagram to illustrate cellular cholesterol homeostasis mechanisms in a stylized, “generic” mammalian cell. The interested reader is referred to the prior review articles cited above [7,8,9] for a more fulsome discussion of mechanisms and the specific molecular “players” involved.

## 2. Functions of Cholesterol in the Retina

Cholesterol is required for retinal development, maturation, and functioning. Cholesterol plays a morphogenic role via the Sonic Hedgehog signaling pathway, which is required for proliferation of retinal neuronal precursor cells [10]. The distribution of cholesterol in the vertebrate neural retina has been demonstrated and discussed elsewhere [8]. Here, we will discuss some known functions of the cholesterol-rich liquid-ordered (L_o_) phase of retinal plasma membrane. The plasma membrane L_o_ phase serves as a critical cell signaling and regulatory hub due to preferential partitioning of several membrane proteins into the L_o_ phase. Proteomic and lipidomic analysis of purified bovine rod outer segment (ROS) disk membranes, ROS plasma membranes, and detergent-resistant membrane (DRM) fractions has provided key insights into the protein and lipid constituents of these separate membrane compartments of photoreceptors [11]. A significant protein constituent of the DRM fraction of bovine ROS plasma membranes is caveolin-1 (CAV-1). CAV-1 also interacts with transducin-α, another DRM-enriched protein, in a cyclodextrin-sensitive, cholesterol-dependent manner [12]. Other ROS DRM fraction residents include glucose transporter-1 (GLUT-1), ROM1, and cyclic nucleotide-gated channel subunits *alpha*-1 and *beta*-1 (CNGα-1 and CNGβ-1, respectively). Very low abundance of rhodopsin (RHO) also was found to colocalize in DRMs, while a majority of RHO and other proteins such as ABCA4 were enriched in the non-DRM plasma membrane fraction of ROS disks [11]. RHO is selectively sequestered to the disk membrane L_o_ phase only when in complex with GTP-bound (activated) transducin-α [13]. These findings independently agree with the known distribution of cholesterol in photoreceptors (PRs) [14,15] and suggest a critical role for cholesterol in efficient PR signal transduction and subsequent synaptic transmission.

The retinal pigment epithelium (RPE) phagolysosomal pathway is necessary for maintaining PR outer segment homeostasis [16]. Cholesterol modulates the functioning of lysosomal acid sphingomyelinase, thereby regulating RPE lysosomal function [17]. Freeze–fracture electron microscopy studies of RPE cells treated with filipin clearly have demonstrated cholesterol enrichment in degradative phagosomes in the RPE [18]. In addition, independent studies have demonstrated a critical role for phagosome membrane cholesterol in phagosome maturation [19,20].

Cholesterol regulates synaptogenesis and synaptic function in the central nervous system (CNS), including the neural retina [21]. Cyclodextrin-mediated depletion of membrane cholesterol increases the lateral motility of voltage-gated calcium channels in cone PRs and bipolar cells [22,23]. We have previously discussed in detail the neuronal dependency on glia-synthesized lipoproteins for synaptogenesis elsewhere [8]. Briefly, results obtained using a retinal ganglion cell (RGC)–Müller glial co-culturing strategy suggested that glia-secreted low-density lipoprotein (LDL) is essential for neuronal synaptogenesis [21]. Ample in vivo evidence also suggests uptake of glia-synthesized LDL by neurons, and such uptake may be necessary for axonal growth [24,25]. This reflects the importance of neuronal–glial interactions in order to maintain neuronal sterol homeostasis and function.

## 3. De Novo Sterol Synthesis in the Retina

Early studies (reviewed in [26]) that demonstrated the ability of the vertebrate retina to synthesize sterols and sterol pathway intermediates de novo (e.g., using radiolabeled mevalonate as substrate) were performed using cell-free homogenates prepared from bovine retinas [27] and, subsequently, with intact bovine retinas in short-term organ culture [28]. However, it was found that the formation of cholesterol was very inefficient in those systems, resulting in accumulation of radiolabel primarily in cholesterol pathway intermediates (sterols and also non-sterol isoprenoids (e.g., squalene)). The first demonstration of the ability of the vertebrate retina to efficiently synthesize cholesterol from de novo substrates was achieved by intravitreal injection of [^3^H]acetate in rats [29] and frogs [30]. Subsequently, it was shown that [^3^H]farnesol could be converted to cholesterol and other sterol products upon intravitreal injection in rats [31]. The first estimates of the absolute rates of cholesterol synthesis, as well as dolichol synthesis (an off-shoot of the mevalonate pathway), were obtained by incubation of frog retinas in vitro in medium containing [^3^H]water [32]. It was concluded from those studies that de novo cholesterol synthesis by the retina was insufficient to account for even the steady-state cholesterol content of retinal ROS membranes, not to mention the cholesterol requirements of the multiple other cell types of the retina. Hence, it became evident, contrary to the “conventional wisdom” at the time, that the vertebrate retina (despite being part of the CNS) is quite unlike the brain, which synthesizes essentially all of its own cholesterol without reliance upon uptake of blood-borne cholesterol (see [33]). More recent studies, using mice fed [^2^H]water and [^2^H]cholesterol, have concluded that 72% of the cholesterol found in the vertebrate retina arises from local de novo synthesis (i.e., by resident retinal cells); by contrast, it was calculated that 97% of cholesterol found in the brain is synthesized de novo by the brain [34] [see Section 4, below].

**Figure 3:**
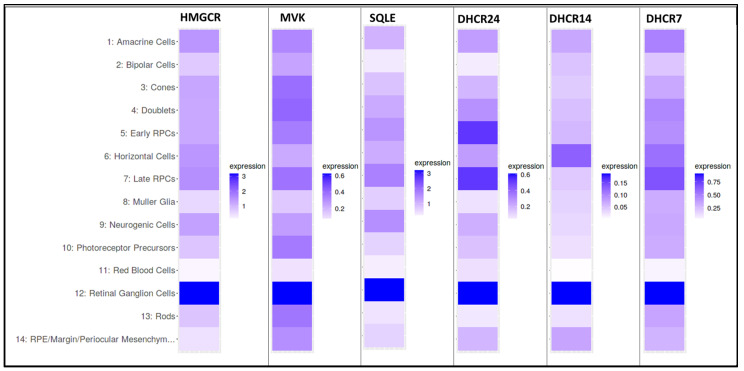
Transcript expression profiles (“heat maps”) of genes encoding key mevalonate pathway enzymes in various retinal cell types and precursors during retinal development (between E11 and P8). These heat maps were generated using a publicly available single-cell RNA-Seq dataset [36] and “Spectacle”, an open-access RNA-Seq data-mining platform [37].

Zheng et al. [14] reported on the spatial distribution of key enzymes involved in cholesterol homeostasis in the human neural retina (also including the RPE and choroid) using qualitative immunohistochemistry, with correlative PCR and qRT-PCR analysis of expression levels of relevant genes in the pathway. Most relevant to de novo cholesterol synthesis, HMG-CoA reductase (HMGCR; the major rate-limiting enzyme of the pathway) was found to localize to multiple histological layers of the retina, most prominently the inner and outer nuclear layers (INL and ONL, respectively), the nerve fiber layer (NFL), and the ganglion cell layer (GCL). Less prominently labeled were the PR inner segment layer, inner and outer plexiform layers (IPL and OPL, respectively), and the RPE. Other molecular players relevant to de novo cholesterol synthesis were found to colocalize with HMGCR, including SREBPs (sterol regulatory binding proteins), INSIGs (insulin-induced gene 1 protein), and SCAP (SREBP cleavage-activating protein). These findings are generally consistent with those of other studies using rat or monkey retinas [35]. Analysis of the protein level distribution of sterol homeostatic machinery in the neural retina suggests expression of the mevalonate pathway in essentially all retinal cell types [14]. Below, we show results obtained by analysis of publicly available single-cell transcriptomic data obtained from the developing mouse retina (between E11 and P8), using a freeware platform (“Spectacle”) (Figure 3); this reveals that RGCs express the highest transcript levels of mevalonate pathway genes [36,37]. These findings, taken together with the neuronal–glial interaction for sterol homeostasis in the mature retina, suggest dynamic changes in cell-specific de novo synthesis of cholesterol in the developing and mature neural retina.

Taken together, there is compelling evidence to demonstrate that the vertebrate retina has the capacity to synthesize cholesterol de novo. While apparently multiple cell types in the retina have this capacity, including PRs, RGCs, RPE cells, and Müller glia, it has yet to be determined, quantitatively, what their individual relative contributions are to the total sterol pool in the retina, either under normal conditions or under conditions involving diseases that impact the structure and function of the retina. Also, although the studies involving [^2^H]water in mice (see above) suggest that 72% of the total cholesterol pool in the neural retina arises from de novo (local) synthesis, it remains to be demonstrated that this holds true for other species, including humans.

## 4. Biological Considerations in Measuring Tissue Sterol Synthetic Rates Using a Radioisotope Approach

Isotopic approaches using deuterated water ([^2^H]water) have been utilized to determine the relative contributions of de novo cholesterol synthesis to the total tissue sterol pool. This approach involves feeding [^2^H]water (typically at 4–10% enrichment), followed by mass spectrometric quantification of [^2^H]labeled cholesterol (or other metabolites) in the tissue of interest [38,39,40,41,42,43]. Here, we discuss several important biological factors that can affect the [^2^H]water-based approach with regard to accurately estimating de novo cholesterol biosynthetic rates in tissues.

The dilution of [^2^H]water administered (in % by vol.) affects its serum enrichment (which needs to be experimentally determined) [43]. Sterol synthetic and turnover rates (often expressed as half-lives) are highly variable between different tissues. For instance, the synthetic rate in liver is extremely high, while the half-life of cholesterol in the brain is extremely long. Therefore, the time period needed for serum equilibration of [^2^H]water should be considered. Typically, serum enrichment of [^2^H]water reaches a plateau rapidly within 6 h [42]; however, the serum enrichment is much lower than the [^2^H]water fraction in the food source [41,42]. Naturally occurring ^13^C and ^18^O isotopes may also be incorporated during cholesterol synthesis in animals fed with unlabeled water, and applying appropriate correction factors for the same is necessary.

The average number of deuterium atoms incorporated per cholesterol molecule is termed “molecular enrichment” (ME). Of the 46 protons in cholesterol (C_27_H_46_O), between 21 and 27 protons are derived from water, in a species-dependent manner [40,44]. Therefore, the dilution of [^2^H]water in the food, its subsequent serum enrichment, and the species and tissue of interest determine the cholesterol isotopomer (isotopic isomer) species distribution. The “molar fraction” (MF) of individual m_n_ species is calculated as m_n_/Σm_n_ [45]. For reliable assessment of sterol synthetic rates, essentially every newly synthesized cholesterol molecule should incorporate at least one deuterium atom. Ideally, the tissue m_o_ species would represent unlabeled cholesterol present in the tissue prior to administration of [^2^H]water, while all sterol molecules synthesized during the experimental period fall in m_n_ species [40]. However, if the ME is low, wherein the dominant m_n_ species contains only one deuterated atom (i.e., n = 1), then a significant proportion of newly synthesized cholesterol during the experimental period would still contain m_o_ species [40]. Hence, a correction factor for unlabeled, newly synthesized m_o_ species should be determined based on the frequency distribution of m_n_ species, to accurately determine the tissue sterol synthetic rates [40]. Measuring retinal sterol synthetic rates using this isotopic approach is very challenging due to the above discussed factors.

## 5. Technical and Biological Challenges in Achieving Tissue/Cell Type-Targeted Inhibition of Cholesterol Synthesis

An alternative strategy to study sterol biosynthetic rates in the tissue of interest, without the use of radioisotopes, involves tissue-specific inhibition of mevalonate pathway enzymes (e.g., HMGCR, SQLE, DHCR14, DHCR7 etc.) and measurement of the rate of accumulation of the relevant enzyme substrate. Such an “enzyme blockade” approach was employed by Keller et al. [46] to measure the absolute rate of cholesterol synthesis in the rat brain (which was validated, in parallel, by monitoring [^3^H]acetate incorporation into brain cholesterol). However, there are no currently available regimens to reliably inhibit mevalonate pathway flux pharmacologically specifically in the retina per se (i.e., not perturbing systemic cholesterol synthesis as well). Intravitreal injection of a statin (inhibiting HMGCR) has been found to cause retinal degeneration and inhibition of the pre-squalene pathway [47,48]. The best available strategy to inhibit cholesterol synthesis is genetic ablation of Kandutsch–Russell or Bloch pathway enzymes. Global knockout of DHCR7 leads to neonatal death, thereby necessitating the development of novel conditional models [49]. The first available conditional DHCR7 model to allow tissue-specific assessment of function was only recently generated [50]. Surprisingly, tissue-specific (liver) deletion of DHCR7 only led to partial (and relatively modest) inhibition of cholesterol synthetic flux [49]. The molecular basis for partial inhibition of flux may be due to functional redundancies between post-squalene pathway enzymes. Evidence for functional redundancy in cholesterol pathway enzymes was first observed between DHCR14 and a nuclear membrane protein called Lamin B receptor (LBR) [51]. Global deletion of DHCR14 only led to partial buildup of 14-dehydrocholesterol, while DHCR14/LBR double knockout led to complete inhibition of sterol synthesis [51]. Interestingly, the sterol reductase domain is fully conserved between DHCR7, DHCR14, and LBR [52]. Similar screening for functionally redundant partners of DHCR7 remains to be reported. Upon identification of other redundant enzymatic partners involved in cholesterol synthesis, retina-specific double knockout of genes encoding them may be necessary for effective targeted and complete inhibition of cholesterol synthesis in the retina.

Preliminary testing of functional redundancy between sterol reductases may be performed using in vitro transcriptional silencing experiments, as well as in silico molecular simulation [53,54,55]. Once candidate redundant reductases are identified, transgenic approaches using conventional Cre-LoxP methodology would be necessary to generate double/triple-knockout mouse lines to achieve successful targeted inhibition of sterol synthesis. Care must be taken in the application of Cre-LoxP transgenic approaches to ensure cell type-specific onset of Cre recombinase activity [56,57]. Cre recombinase lines often exhibit unanticipated (and unintended) germline recombination, as well as “leaky” (ectopic) expression in non-target cell types [56,57,58,59]. We have recently described a simple workflow approach using a fluorescent reporter mouse line to maintain retinal cell type-specific Cre mouse lines, such as RPE65-Cre, CRX-Cre, GFAP-Cre, and Rho-iCre [60,61,62,63,64]. We propose that reporter-verified, cell type-specific, double-gene knockout is the only available tractable and reliable method to investigate the role of cellular de novo synthesis of sterols.

## 6. Sterol Uptake by the Retina

Unlike the capillaries that constitute the main elements of the blood–brain barrier, which excludes circulating lipoproteins [33] from entering the brain, the choroidal vasculature has a fenestrated capillary network (the choriocapillaris) [65] that can allow passage of blood-borne lipoproteins from the blood to the RPE—the cellular interface between the choroidal blood supply and the outer neural retina. The basal surface of the RPE is populated by multiple lipoprotein receptor subtypes, which can facilitate uptake of those lipoproteins [66,67,68,69,70,71]. The ability of cholesterol carried by blood-borne lipoproteins to be taken up by the retina was first demonstrated by Tserentsoodol et al. [35] in rats [see also below]. In a series of unrelated studies, the ability of diet-derived cholesterol to alter the steady-state sterol composition of the rat retina was demonstrated [72], again implicating blood-borne lipoprotein uptake as a significant factor contributing to cholesterol homeostasis in the retina. The subsequent study by Mast et al. [34], referenced above, provided important additional quantitative information in this regard, where it was estimated that nearly 30% of the sterol content of the mouse retina arises from uptake of cholesterol from the blood. More recent studies, performed using mice [73] and hamsters [74,75], have validated and extended those results.

The Tserentsoodol et al. [76] study employed intravenously injected human-derived LDL “doped” with cholestatrienol (cholesta-Δ^5,7,9(11)^-trien-3β-ol), a naturally fluorescent cholesterol analog, to monitor lipoprotein uptake by the rat retina, as a function of time post-injection, using confocal fluorescence microscopy analysis of ocular tissue sections. Fluorescence first appeared in the choroid and RPE, then sequentially in the outer retina (PR layer), and then spread to the inner retinal layers to the vitreoretinal interface. This suggested intraretinal transport of circulating lipoprotein-borne cholesterol that was initially taken up by the RPE. A companion study by the same group validated that such a mechanism is present in the neural retina, primarily involving HDL (as the vehicle for cholesterol transport) and a host of other molecular players (e.g., ABCA1, scavenger receptors (SR-I and SR-III), CD36, CETP (cholesteryl ester transfer protein), and LCAT (lecithin-cholesterol acyl transferase)) [76]. These findings were generally corroborated (and extended significantly) by Zheng et al. [76].

Hence, there is compelling evidence to demonstrate the capacity of the vertebrate retina to take up exogenous (extraretinal) cholesterol from blood-borne lipoproteins, using a receptor-mediated mechanism. How that pool of cholesterol is utilized by the various cell types of the retina, what percentage of that pool is taken up by each retinal cell type, and exactly how it becomes distributed throughout the different histological layers of the retina remain to be elucidated.

## 7. Sterol Efflux from the Retina

Having considered the “supply side” of cholesterol homeostasis in the retina, we now turn our attention to how the retina gets rid of excess cholesterol. Cholesterol typically undergoes enzymatic hydroxylation and oxidation (by CYP27A1 and CYP46A1) to generate relatively less hydrophobic oxysterols which may be easily effluxed from the cell [77]. The predominant hydroxy- and oxysterols generated by CYP27A1 and CYP46A1 include 24-, 25-, and 27-OH-cholesterol [77]. Other enzymatic players involved in generating oxysterols are TSPO (translocator protein) and NADPH oxidases [78]. Cellular stressors such as Fenton reaction products can also contribute to the generation of oxysterols [79]. The primary players involved in cellular sterol efflux as HDL particles are ABCA1 and ABCG1 in most tissues, including the retina, with the exception of the brain, which utilizes ABCG1 and ABCG4 for sterol efflux (reviewed in [8,33]). ABCA1/ABCG1 sequesters cellular oxysterols into ApoA1-containing HDL particles and thereby regulates HDL formation and cholesterol efflux from cells. The expression of these ABC transporters is regulated by LXRα and LXRβ, in rod PRs and RGCs, respectively (reviewed in [8]). ABCA1 was found to be enriched in the RGC layer, RPE, as well as PR inner segments [76]. Concomitant deletion of ABCA1 and ABCG1 from rod PRs led to the accumulation of cholesterol, 24-, 25-, 27-OH-cholesterol [80]. Similarly, ABCA1/G1 deletion in RPE cells also leads to lipid efflux defects and retinal dystrophy [81]. Global concomitant deletion of sterol-oxidizing enzymes CYP27A1 and CYP46A1 causes retinal dysfunction as early as PN 3 months and is characterized by a significant increase in retinal cholesterol content [82]. Together, these studies suggest a critical role for lipid efflux mechanisms in appropriate maintenance retinal structure and function as well as to overall retinal cholesterol homeostasis.

## 8. Strategies to Measure Sterol Uptake and Efflux Rates in the Neuronal Retina

Investigation of sterol synthesis using an isotope-based strategy is challenging (as discussed above), since it requires the use of radiolabeled precursors, such as deuterated or tritiated water, acetate, or mevalonate. We have previously demonstrated, using a pharmacologically induced model of Smith–Lemli–Opitz syndrome (SLOS), that dietary cholesterol (2% *w*/*w* in the food) was sufficient to almost fully replace the retinal sterol pool [72,83]. However, the rate of replacement of retinal sterols in that model may not be truly reflective of retinal cholesterol turnover rate, due to the significant buildup of relatively less hydrophobic oxysterols. However, while measurement of retinal cholesterol uptake and efflux rates using an isotopic approach is technically feasible, unfortunately it tends to be prohibitively expensive. The strategy involves dietary supplementation with deuterated cholesterol (d5 or d7, at 1% *w*/*w*). Based on the sterol turnover window observed in the aforementioned SLOS study, animals may need to be placed on this diet for about one month. Time-dependent enrichment of labeled cholesterol in the retina (at 4 or 5 different time points) will be truly reflective of the sterol uptake profile in the neural retina of the specific species of interest. A cohort of such special chow-fed animals then may be weaned, and the time-dependent loss of isotope-labeled cholesterol would be reflective of the retinal cholesterol efflux rate.

## 9. Conclusions

From the foregoing discussion, it is clear that the fundamental features of the cholesterol homeostasis process in the vertebrate retina are known, at least in broad terms. That knowledge rests upon the published outcomes of animal-based (mostly mice and rats) in vivo studies as well as in vitro animal cell culture and cell-free tissue homogenate metabolic studies. However, there remains to be learned further details, at the molecular and cellular levels, as well as the system level, about the process. The results obtained to date from lab-based experimental studies are generally consistent with what is known about systemic and brain cholesterol homeostasis in humans; however, it remains to be confirmed that those same findings are quantitatively applicable to human retinal cholesterol homeostasis as well as more generally across vertebrate species. While there are several known human hereditary diseases caused by cholesterol biosynthetic defects, they are all recessive and, thankfully, rare, and none of them are non-syndromic (i.e., all bodily tissues are affected, not specifically the retina) [2,84]. While pharmacological and dietary supplementation approaches have been tried as therapeutic interventions for clinical management of patients afflicted with such diseases, in general, those approaches have not proven to be widely effective [85,86,87,88]. With regard to human diseases that involve disruption of cholesterol homeostasis that involve structural and/or functional abnormalities in the retina, it is more often the case of having too much cholesterol (and its esters and oxidized by-products), i.e., deposition and failure to efficiently remove excess cholesterol-rich deposits, rather than local defective de novo synthesis of cholesterol [89,90]. The use of statins as a treatment option for AMD has been tried and debated for many years (see [91,92,93]), with conflicting results and without current definitive resolution. Also, very recently it has been proposed that different biological processes may underlie the formation of specific types of cholesterol-rich deposits associated with AMD (e.g., drusen vs. sub-drusenoid deposits (SDDs)), resulting in different disease states); hence, different therapeutic intervention strategies may be required to resolve those distinct pathologies [94]. More effective therapeutic interventions in such diseases may require a combination of pharmacological, dietary, and gene therapy-based interventions, guided in part by data-mining approaches [95] and application of refined pharmacogenomics [96] as well as use of more recently developed and emerging pharmaceuticals for improved lipid management [97].

## Data Availability

No new data were created or analyzed in this study. Data sharing is not applicable to this article.
